# P-1725. Computed tomography findings in biopsy proven invasive pulmonary mold infection: solid organ transplant versus hematologic malignancies (A 22-Year review)

**DOI:** 10.1093/ofid/ofaf695.1896

**Published:** 2026-01-11

**Authors:** Hyeon Mu Jang

**Affiliations:** Asan medical center, Seoul, Seoul-t'ukpyolsi, Republic of Korea

## Abstract

**Background:**

Computed Tomography (CT) patterns of pulmonary invasive mold infection (PIMI) can vary in a wide spectrum, depending on the profile of underlying diseases and immunocompromised status. Thus, we systematically investigated more than 20 years of biopsy proven pulmonary invasive mold infection and compared its CT findings between patients with a history of solid organ transplant (SOT) and hematologic malignancy (HM).

**Table 1.**
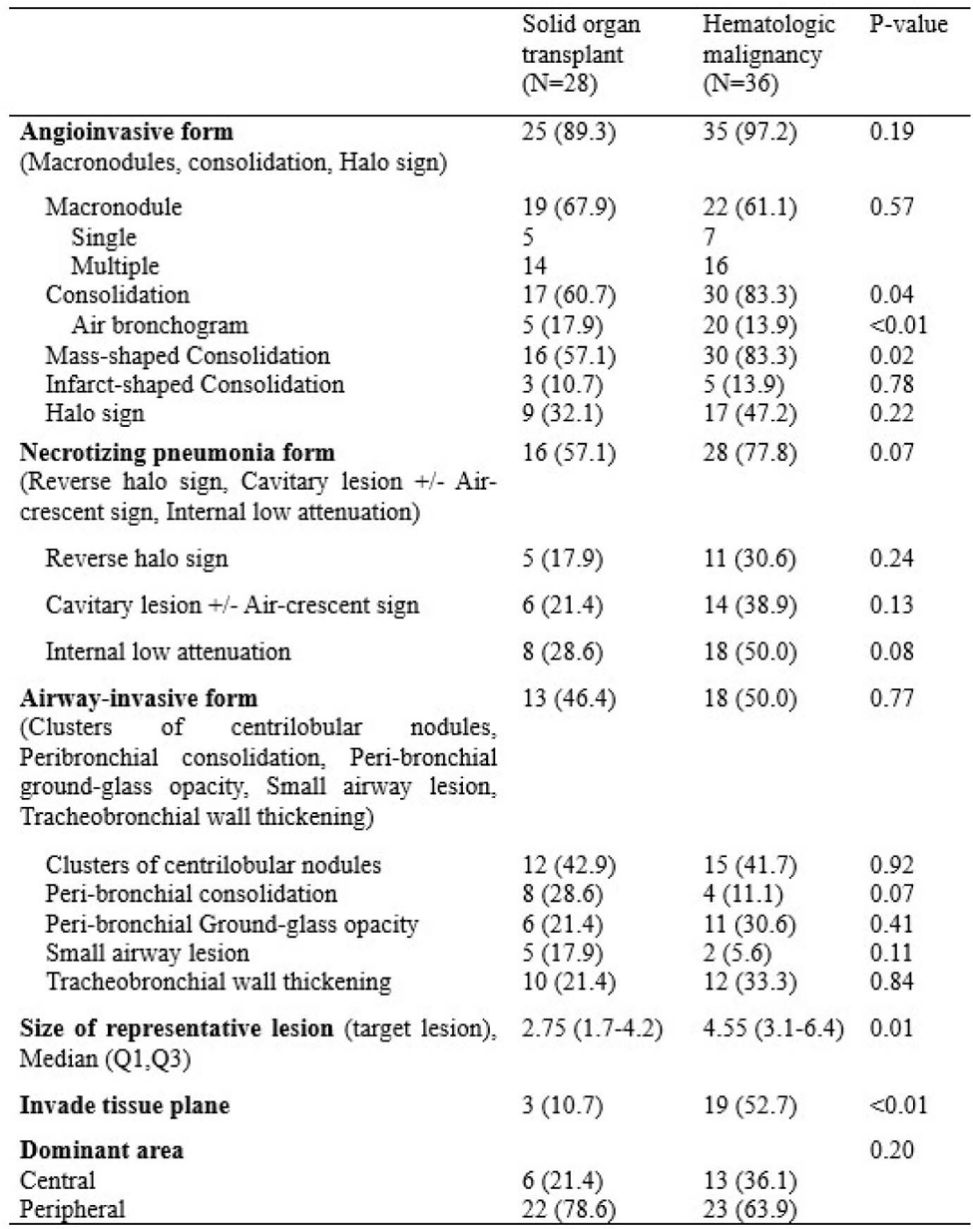
Comparison of computed tomography findings of pulmonary invasive mold infection between patients with a history of solid organ transplant and hematologic malignancy

**Methods:**

The medical records were retrospectively reviewed, of adult patients (aged ≥18 years) from January 2003 to June 2024 who met the criteria for proven PIMI according to the Mycosis Study Group (EORTC/MSGERC) Guideline. Tissue plane invasion is defined when a representative lesion traverses tissue planes including the lung fissures, diaphragm, chest wall, and a pleura rather than being contained in a lung lobe.

**Results:**

A total of 64 patients, including 28 patients with a history of SOT and 36 patients with HM, were analyzed. The underlying lung disease was more common in SOT (12/28 [42.9%] vs 3/36 [8.3%]; p< 0.01) whereas prolonged neutropenia (< 0.5 × 10^9^ neutrophils/L [< 500 neutrophils/mm^3^] for >10 days) was more common in HM. Mucormycosis were more frequent in HM than SOT (4/28 [14.3%] vs 18/36 [50.0%]; p < 0.01). When compared to SOT, PIMIs in patients with HM had significantly higher tendency to invade the tissue plane (3/28 [10.7%] vs 19/36 [52.7%]; p < 0.01). The size of the representative lesion was much bigger in HM (27.5mm [17.0-42.0] vs 45.5mm [31.0-64.0]; p 0.01). Consolidation was significantly more common in HM (17/28 [60.7%] vs 30/36 [83.3%]; p 0.04).

**Conclusion:**

The radiological features of PIMI differed between SOT recipients and patients with HM. In patients with HM, PIMI were more likely to invade the tissue plane, to have bigger representative lesions, and to show consolidation. More studies are needed to evaluate CT characteristics with different types of immunocompromised status.

**Disclosures:**

All Authors: No reported disclosures

